# Enhancing the Salt Frost Durability of Concrete with Modified Epoxy Composite Coating

**DOI:** 10.3390/ma18040737

**Published:** 2025-02-07

**Authors:** Lu Cong, Yanchao Wang, Xuekai Gao

**Affiliations:** 1 Department of Civil Engineering, Shanxi University, Taiyuan 030006, China; conglu800010@sxu.edu.cn; 2 Transportation Industry Key Laboratory of Highway Construction and Maintenance Technology in Loess Area, Shanxi Province Transportation Technology Research and Development Co., Ltd., Taiyuan 030006, China; gocek@alu.hit.edu.cn

**Keywords:** cement concrete, surface coating, modified epoxy composite, salt frost resistance, microscopic mechanism

## Abstract

A durable and easy-to-operate treatment, modified epoxy composite coating (MECC), was proposed in this study as a potential alternative to traditional epoxy resin protectants to enhance the protection of concrete structures. This new material consists of epoxy resin as the base material, dimethyl carbonate as the solvent, and modified amines and polyaniline as a composite curing agent that reacts with epoxy resin to form a film over the surface of concrete, thus protecting concrete structures from surface cracking, peeling, and spalling when exposed to chloride. Salt frost resistance tests indicated that MECC specimens had lower water absorption and much higher salt frost resistance. Compared with non-coating (NS) specimens, after 200 freeze–thaw cycles, the relative dynamic elastic modulus (RDEM) was 21.62% higher, and the mass loss was merely 19.14% of that of the NS specimens. Better performance was achieved as compared with ordinary epoxy resin coating (EC) and silicate coating (SC) too. After 120 days of erosion in 10.0% NaCl, the coating could effectively prevent environmental liquids and chloride from intruding through the cracks. The reason behind the increased salt frost durability is that treatment with MECC improved the internal structure of concrete and made its surface dense enough to prevent the intrusion of environmental liquids and chloride. Under repeated freezing and thawing, the degree of chloride-induced damage and the icing pressure inside the concrete were greatly reduced. This relieved the frost damage inside the concrete and elongated the service life of the concrete.

## 1. Introduction

Chloride erosion into concrete, as a highly hazardous aggressive media damage and one of the major factors compromising the durability of concrete, is reported to cause enormous economic losses every year [1]. For concrete structures on highways and bridges, surface coating is the most common way of protection, especially in cold areas. Coating protection can minimize physical and chemical erosion damage to concrete structures caused by snow-melting agents and deicing salts, and prevent them from deteriorating prematurely due to durability problems [2,3].

For economic considerations, the deicing salts used on highways and bridges are primarily composed of chloride; however, the chloride erosion constitutes one of the main contributors to damage on highways and bridges. Chloride ions are detrimental to concrete structures on highways and bridges in two ways [4]. On the one hand, they crystallize into salt inside cement concrete and expand, causing cement concrete to crack. Chloride ions react with calcium hydroxide in cement. Due to the presence of chloride ions, the CaCl_2_ generated is more soluble than Ca (OH)_2_. As a result, the osmotic pressure of the salt solution causes the water inside the concrete to migrate toward the external surface, causing the concrete to expand and crack more rapidly. When the concentration of CaCl_2_ in the concrete is high (>15%), it can combine with CaO into very tiny, needle-like crystals in the liquid phase, which, when sufficiently accumulated, can lead to expansion and corrosion of the concrete [5]. Microscopic studies have also demonstrated that chloride ions can penetrate into concrete and damage the internal structure of gel, while the presence of high concentrations of chloride can intensify the dry shrinkage of concrete [6,7]. On the other hand, the use of snow-melting agents and deicing salts causes an increase in the chloride concentration on the surface of highways and bridges. With a small radius and high activity, chloride ions can easily penetrate the passivation film of the concrete and cause the reinforcement to rust [8]. When the chloride content in the solution is high or after repeated dry/wet cycles, more salt will be brought into the concrete. As a result, the invasion of chloride ions will produce high crystallization pressure in the concrete pores [9] and promote corrosion-induced damage to the surrounding concrete [10], which causes the concrete surface to spall, crack, or crumble, resulting in shorter service life of the concrete structure than expected by design.

In order to increase the service life of concrete structures, research has focused on green, low-carbon, and coating-based protection for concrete materials [11,12]. The purpose is to obtain better concrete performance, especially surface protection and maintenance [13]. Epoxy coatings, comprising primarily of epoxy resin, have been developed and widely applied [12]. The idea behind this treatment is to form a tight physical protection over the concrete surface [2], so as to prevent aggressive media from coming into the interior of the concrete and increase its durability [3,4]. Today, epoxy resin coatings are still the most widely used, most widely applicable, and the most important corrosion protection in the world. Surface coating can form a dense watertight layer over the concrete surface and prevent the intrusion of external corrosive media. Diamanti et al. [14] investigated the effect of polymer-modified cementitious coatings of two different polymer-to-cement ratios on the water permeability of concrete and found that epoxy resin coatings as a physical barrier are well able to minimize water intrusion into concrete. Unfortunately, the durability of epoxy resin-cured materials is heavily restricted by their high internal stress, low toughness, ready degradation under high temperatures, and poor wet stability [3,12]. To solve these problems, Fei Li [15] prepared a water-based hydrophobic slurry by combining two cost-effective, highly available materials, epoxy resin and octadecylamine (ODA), and used it as a binder in o-concrete. They eventually created a superhydrophobic concrete composite with a water contact angle of 156° and a sliding angle of 8°. Liu [16] prepared a nano-composite anti-corrosion filler with a mass ratio of CeO_2_: GO = 4:1 by hydrothermally growing cerium oxide on the surface of graphene oxide. The corrosion resistance of reinforced concrete in the marine environment was effectively improved. Guo et al. [17] prepared a novel TiO_2_-graphene-modified epoxy resin as a coating for ordinary Portland cement (OPC) concrete. It was found that adding given amounts of TiO_2_-graphene nanofiller (0.3% and 0.5%) greatly improved the impermeability of chloride (as reflected by capillary adsorption and chloride permeability). Silicone, which features Si-O bonds with high bonding energy [18], has been proven to improve the thermal stability, weather resistance, and brittleness of epoxy resin [18,19]. Furthermore, as silicone has low surface free energy, its application over the surface of epoxy resin can enhance the water and oil resistance of the material [20]. Zheng et al. [21] prepared a solvent-free, epoxy heavy-duty anticorrosive coating composed of modified epoxy resin, reactive toughening diluent, and other additives, and this significantly enhanced the material’s resistance to salt spray and corrosion. Qu et al. [22] prepared coated concrete specimens by adding nano-SiO_2_ and nano-TiO_2_ into epoxy resin coating and subjected them to accelerated aging under ultraviolet irradiation, followed by accelerated carbonation, or Coulomb electric flux test. The long-term carbonation and chloride erosion resistance of the coated concrete were significantly improved. Fernández-Álvarez et al. [23] discovered that SiO_2_ nanoparticles can increase the contact angle of cryptic epoxy resin coatings and enhance their resistance to permeation and chloride ion erosion. So far, research has covered many types of anticorrosive coatings, including solvent-based and solvent-free epoxy anticorrosive coatings [3]. Dorado et al. [24] demonstrated that the most promising adhesion values can be delivered by adding 3 wt% of the synthesized Fe_2_O_3_ nanoparticles into a graphite carbonaceous matrix; the nano-modified coating displayed much greater resistance to chemical corrosion and surface wear. However, there are some problems pending further studies, such as the cost of nanoparticles and silicone as a modifier and the weather resistance of rubber-modified epoxy resin [2,3,14].

Engineering practices in northern China have proved that epoxy resin coatings for highway and bridge concrete structures lack sufficient freeze–thaw durability [25,26] since aggressive media like hydroxide and chloride solutions will penetrate through the coating and react with concrete, resulting in gradual failure of the physical protection [6,27]. Within the second to third service year of the epoxy resin coating, surface spalling and structural strength degradation will occur to the structure as a result of freezing and thawing processes involving chloride [28,29]. This greatly shortens the maintenance cycle and increases the maintenance workload, bringing additional labor and material costs [30,31]. It is therefore necessary to improve epoxy resin products and enhance the durability of concrete coatings, especially in seasonally frozen areas, where highway concrete structures are exposed to erosion induced by snow-melting agents.

In this study, we present a modified epoxy resin coating material and an improved epoxy resin protectant to provide longer surface protection and enhance the durability of concrete structures on highways and bridges. Dimethyl carbonate was used as the solvent, while modified amines/polyaniline served as a composite curing agent that reacts with epoxy resin to form a film on the concrete surface. Through laboratory tests, the effect of this modified coating on improving the freeze–thaw resistance of concrete is verified. A freeze–thaw test was used to determine the mass loss and relative dynamic elastic modulus (RDEM) of cement concrete specimens in 3.50 wt% NaCl and water, as well as their chloride ion permeation resistance and saturated water absorption. We then measured the pore structure parameters of the specimens using mercury intrusion porosimetry (MIP) and industrial computed tomography (CT). Subsequently, we analyzed the mechanism underlying the salt frost durability of the MECC specimens from a micropores perspective.

## 2. Materials and Methods

### 2.1. Materials

Table 1 presents the chemical composition of the cement. The fine aggregate consisted of natural river sand (fineness modulus = 2.7 and bulk density = 2710 kg/m^3^). The coarse aggregate was 5–10 mm and 10–20 mm continuously graded. Table 2 provides the mixing ratio of the cement concrete specimens. The concrete had a w/cratio of 0.40 and a 28-day compressive strength of 46.0 MPa. The cement used was P.O.42.5 Portland cement(specific area = 300 m^2^/kg and consistency = 22.5%), produced by Hangzhou Haishi Cement Co., Ltd.(Hangzhou, Zhejiang, China). The water reducer was a high-efficiency polycarboxylate water reducer, produced by Shanxi Transportation Research Institute Group Co., Ltd. The test water used was tap water. Three concrete surface modifiers were used, including silane, ordinary epoxy resin, and the modified epoxy resin, provided by Shanxi Transportation Research Institute Group Co., Ltd. (Taiyuan, Shanxi, China) Modified composite part A was prepared with the following mixing ratio: epoxy:mica:glass flake:mica iron oxide:promoting agent:zinc phosphate:penetrant:solvent = 100:15:20:10:10:5:3:10, with dimethyl carbonate as the solvent. The layered structure of mica and sheet structure of mica iron oxide form the excellent barrier properties of MECC, capable of preventing the dispersion of corrosive media (Cl^−^/SO_4_^2−^). By using glass flake, which functions similarly, it can aid to forming complicated and flexural penetrating and dispersing pathways inside the MECC. This prevents corrosion of the matrix, as the corrosive medium is unable to diffuse through the flexural dispersing pathways. The zinc phosphate can facilitate the formation of a compact and indissolvable protective film in the MECC, providing chemical anti-rust properties. The promoting agent and penetrant play a main role in assisting the modified epoxy resin to be penetrated deeper into the surface structure, resulting in a more compact coating. Modified composite part B was prepared with the following mixing ratio: heterogeneous polyaniline:N-methylpyrrolidone (NMP):modified amine curing agent = 20:100:30. Part A and Part B were mixed in the mixing ratio of A:B = (2.5–3.5):1 to produce the desired modified composite epoxy resin material for concrete surface protection. The epoxy resin anticorrosive material was a film-forming transparent emulsion with an effective content of 81.0% and a coating thickness of 2.8–4.0 mm. The silane was a silane-impregnated penetration coating composed mainly of high-purity isobutyl silane, with a silane content of 98.9%. The chloride solution for preparing the environmental solution consisted of sodium chloride powder (AR, 99%), produced by Shanghai Aladdin Biochemical Technology Co., Ltd., (Pudong New District, Shanghai, China).

### 2.2. Experimental Design and Sample Treatment

**Figure 1:**
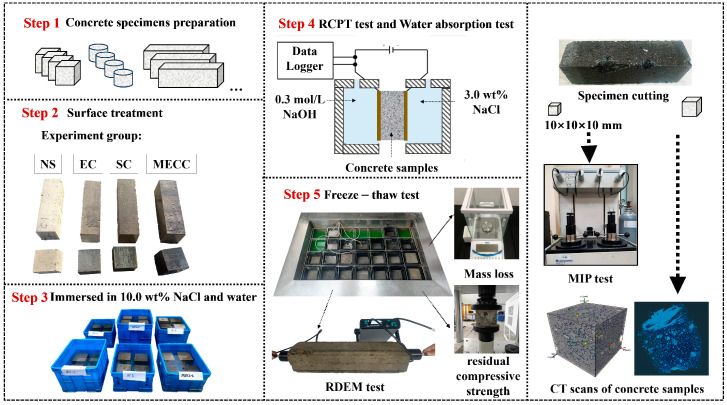
Diagram of test procedure.

As shown in Table 3, the concrete specimens were treated with three types of surface coatings, with detailed treatment procedures provided. The cement concrete specimens were demolded 24 h after they were formed and then cured at 20 ± 3 °C and a relative humidity of 90 ± 3% for 28 days. Subsequently, all the specimens were treated as specified in Table 3. The epoxy resin, silane, and modified epoxy resin were prepared one day before specimen treatment and thoroughly mixed using an electric stirring rod. To control the influence of coating thickness on the experimental results, a plastic brush was used to sequentially apply the coating to all six sides of the concrete specimens, which prevents waste of uncured coating material. The coating specimens were then cured for 48 h at 25 °C and a relative humidity of 65% before the test. The test procedures are illustrated in Figure 1, and detailed test methods are described in the Section 2.3.

### 2.3. Test Methods

#### 2.3.1. Salt Frost Resistance Test

In addition, following the “Standard for test methods of long-term performance and durability of ordinary concrete” (GB/T50082-2009) [32], we applied a new test procedure and tested the cement concrete specimens by erosion and freezing and thawing, as shown in Figure 1. First, three specimens per group (100 mm × 100 mm × 400 mm) were treated and then eroded in 10.0 wt% NaCl solution for 120 days. Then, we took the specimens out, tested their initial ultrasonic velocity, and then subjected them to rapid freezing and thawing for 200 cycles in 3.5 wt% NaCl solution according to the main snow-melting agent used in highway projects [9,27]. The rapid freeze–thaw test was conducted at the maximum and minimum temperatures of 5 °C ± 2 °C and −18 °C ± 2 °C, with each cycle lasting 4 h. After every 25 cycles, we tested the mass loss of the specimen, and after every 40 cycles, the relative dynamic elastic modulus (RDEM) was calculated by ultrasonic pulse velocity measurements [33]. The RDEM ($P_{c}$) for concrete specimens was determined using Equation (1), where e and $V_{0}$ represent the ultrasonic pulse velocity, respectively. After 200 freeze–thaw cycles, the samples were taken out and cut into 100 mm cubes, and their residual compressive strength was measured.

(1) $$P_{c}\left(\%\right)=\frac{V_{c}^{2}}{V_{0}^{2}}\times 100\%$$

#### 2.3.2. Water Absorption

Impermeability is associated with environmental erosion resistance. The water absorption of the cement concrete specimens (150 mm × 150 mm × 150 mm) was measured according to the “Standard for test methods of concrete physical and mechanical properties” (GB-T 50081-2019) [33]. The specimen to be tested was completely immersed in water, with the top surface of water 5.0 ± 0.5 mm above the top surface of the specimen. First, we measured the surface dry mass of the specimen (m_s_). Each specimen was immersed in water for a minimum of 48 h and until the mass change over two 24 h intervals was less than 0.2% of the larger value. After the measurement, we dried the specimen in a 105 ± 5 °C drying oven and then measured the mass of the dried specimen (m_d_). Each specimen was dried for a minimum of 48 h and until the mass change over two continuous 24 h intervals was less than 0.2% of the smaller value. The concrete water absorption W_r_ was calculated by Equation (2):

(2) $$W_{r}=\left[\left(m_{g}-m_{0}\right)/m_{0}\right]\times 100\%$$

#### 2.3.3. Rapid Chloride Permeability Test

We tested the permeability of chloride ions in the coating specimens by the rapid chloride permeability test (RCPT) [32] and observed the surface morphology of the specimens after a 120 d long-term immersion. RCPT specimens, sized Φ100 mm × 50 mm, was immersed in 10.0 wt% NaCl solution. We used a YC-RCM concrete chloride ion migration coefficient tester for this purpose. When switching the test instrument on, the solutions in the vessels at the sides of the system were 0.3 mol/L NaOH (positive pole) and 3.0 wt% NaCl (negative pole). Under an applied electric field of 60 V, we measured the current flowing through the specimen every 30 min. The test lasted 6 h. We then calculated the total electric flux of the specimen according to Equation (3), where $Q_{s}$(C) denotes the electric flux through the Φ95 mm×50 mm concrete specimens and $Q_{x}$ (C) denotes the electric flux through the Φ100 mm × 50 mm concrete specimens. Table 4 gives the RCPT evaluation criteria for chloride ion permeability.

(3) $$Q_{s}=0.95^{2}\times Q_{x}$$

#### 2.3.4. Microstructure Testing

The pore distribution and permeability variation of surface samples were examined by MIP, using a Micromeritics AutoPore V 9620 analyzer (Micromeritics Instrument Ltd., Norcross, GA, USA). The samples (10 mm × 10 mm × 10 mm) were taken from the surface layer of the concrete for the freezing and thawing test. We then investigated the freeze–thaw damage of the concrete internal structure by CT scanning, using an EcoVision Y. CT Precision high-accuracy CT system (The European X-Ray Free-Electron Laser Facility, Schenefeld, Germany). After 120 d of chloride immersion and 200 freeze–thaw cycles, the damaged concrete samples were cut with a cutting machine to remove the internal part, and then they were resized into 40 mm × 40 mm × 40 mm cubes.

## 3. Results

### 3.1. Salt Frost Resistance

**Figure 2:**
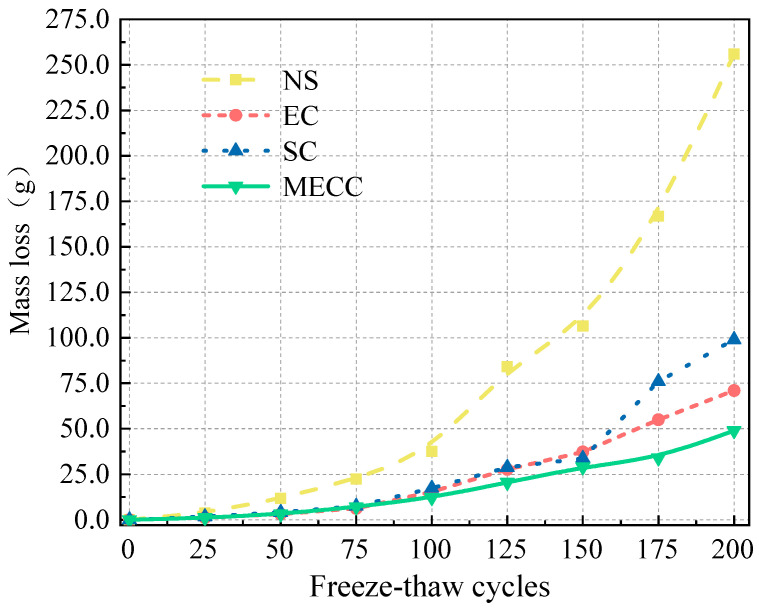
Mass loss under different freeze–thaw cycles.

**Figure 3:**
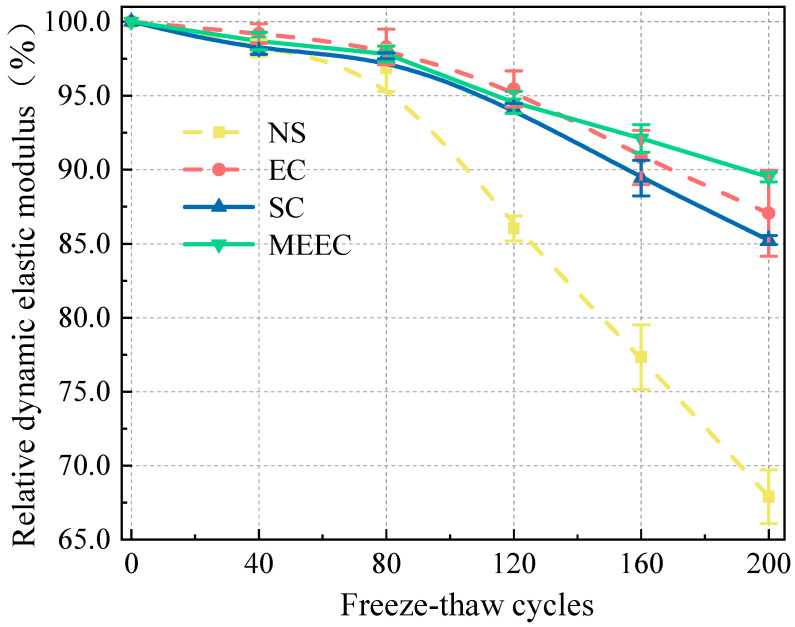
RDEM under different freeze–thaw cycles.

**Figure 4:**
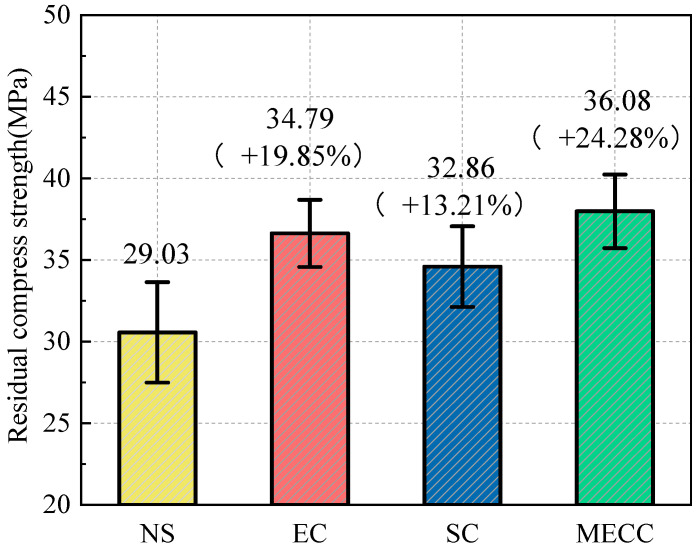
Residual compressive strength after 200 freeze–thaw cycles.

**Figure 5:**
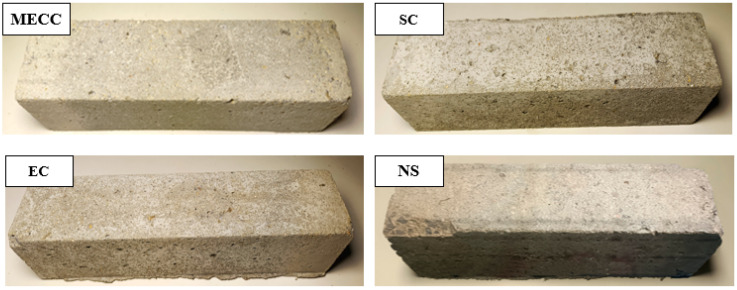
Surface morphology of concrete specimens after 200 freeze–thaw cycles in 3.5 wt% NaCl solution.

To investigate the salt frost resistance of MECC-treated concrete, we analyzed the frost performance of concrete specimens in terms of RDEM, mass loss, and residual compressive strength [34]. Figure 2 and Figure 3 compare the mass loss and RDEM of different specimens under different numbers of freeze–thaw cycles. Figure 4 and Figure 5 compare the residual compressive strength and surface morphology of the specimens after 200 freeze–thaw cycles. Overall, MECC could enhance the salt frost resistance of concrete structures and enhance the salt frost durability of concrete structures better than ordinary epoxy resin and silane. Under the same freeze–thaw conditions, the MECC specimens had the lowest mass loss and RDEM loss and the highest residual compressive strength, meaning that they had the least freeze–thaw damage. From the surface morphology analysis, the NS specimens had the worst surface damage, with exposed coarse aggregate at the corners. The surface of the SC and EC specimens had many holes, although fewer holes were observed in the EC specimens. Both groups showed slight slagging on the surface, but no aggregate spalling or damage was detected. The MECC specimens maintained good surface morphology and uniform surface color, with limited pore exposure and no aggregate spalling.

The NS specimens ranked the worst for all three evaluation criteria, with the largest average mass loss (256 g). None of the coating specimens suffered aggregate spalling except some surface damage for the SC and EC specimens. The surface morphology of the MECC specimens was well intact. The average mass loss of the coating specimens was 99 g (SC), 71 g (EC), and 49 g (MECC), which were 38.67%, 27.73%, and 19.14% of that of the NS group. Throughout the freeze–thaw test, the surface damage of the NS specimens intensified after 50 freeze–thaw cycles, and their mass loss became obvious. For the coated specimens, due to the protective surface coating, the mass loss took place more mildly and slowly. The mass loss did not intensify significantly for the SC and EC groups until after 150 cycles; whereas the mass loss rate of the MECC group remained almost unchanged throughout the test, suggesting that MECC provides a reliable surface protection for concrete structures.

The RDEM variations of the specimens were analyzed. After 200 freeze–thaw cycles, the RDEM values of the four groups of specimens were 67.89% (NS), 85.24% (SC), 87.06% (EC), and 89.51% (MECC). Surface coating increased RDEM by 17.35%, 19.17%, and 21.62%. NS also showed the largest RDEM loss among all groups, followed by SC, EC, and MECC, which was consistent with what has been observed for mass loss. Silane can improve the salt frost performance of concrete, but SC did not perform as well as EC or MECC in the next half of the freeze–thaw test. The EC group showed roughly the same surface damage as the SC group and its RDEM loss was further reduced. Notably, color unevenness was observed on the surface of the EC and SC specimens, possibly because the surface coating on these two groups of specimens was not uniformly distributed on the concrete substrate. Under freeze–thaw cycling, the protection in some areas disappeared, leading to premature damage to the concrete surface. Even though the damaged areas were not at the corners, erosion of these areas could result in local damage while leaving the other areas intact. However, further studies will have to be conducted to confirm this possibility. Compared with the other three groups, after 200 rapid freeze–thaw cycles, the RDEM loss in the MECC group was reduced by 21.62% (NS), 4.27% (SC), and 2.45% (EC), and the specimens had a longer MECC decay cycle. According to the specified criteria for frost damage, this signifies that the samples with MECC treatment can withstand more freeze–thaw cycles before reaching the frost damage limit.

Residual compressive strength is another important criterion for evaluating the degree of internal damage of concrete specimens [34,35]. As shown in Figure 4, the residual compressive strength of the NS specimens was 29.03 MPa, which was about 63.10% of the initial level. For the SC, EC, and MECC specimens, the residual compressive strengths were 32.86 MPa, 34.79 MPa, and 36.08 MPa, respectively, representing 13.21%, 19.85%, and 24.28% increases compared to the NS specimens. These results confirm that surface coatings can effectively enhance the frost resistance of concrete. The MECC samples had better frost resistance, which is encouraging for the continued research and development of this new type of surface treatment.

### 3.2. Water Absorption Reduction

**Figure 6:**
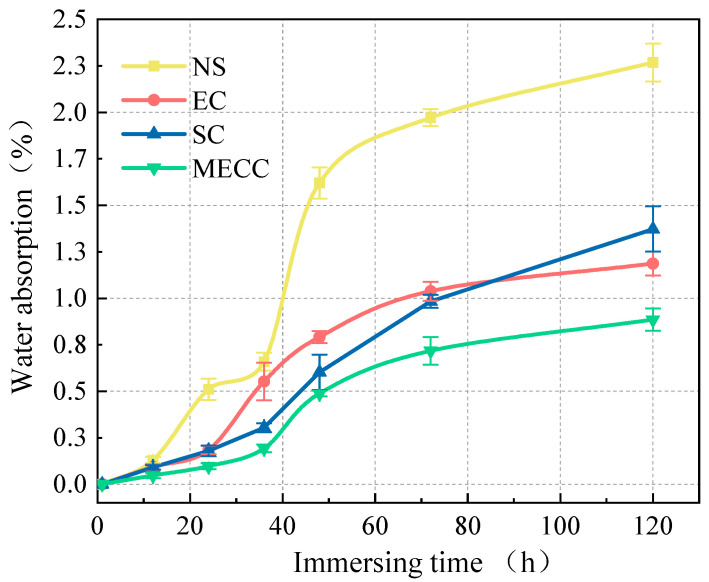
Relationship between water absorption rate and immersion time.

As a physical barrier for protecting cement concrete, one of the roles of a coating is to largely reduce water intrusion into the interior of concrete, thereby reducing icing pressure and consequently the freeze–thaw damage under repeated freezing and thawing [3,36]. The water absorption test is to detect how different types of coating prevent water intrusion into the interior of concrete. Figure 6 compares the water absorption test results of different specimens. When the specimens were immersed in water, the water absorption of the NS specimens increased steeply over the first 48 h. As the immersion time further increased, they slowed down water absorption and became saturated at 2.16%. For the coating samples, the water absorption was much lower, as previously expected, being 1.32% (SC), 1.13% (EC), and 0.93% (MECC). The MECC specimens had the lowest water absorption, which was 56.94% lower compared with the NC specimens. The EC specimens showed roughly the same water absorption increase as the MECC specimens; they absorbed water at a slowly increasing rate over the first 36 h and were nearly saturated when immersed for more than 48 h. Notably, the water absorption of the SC specimens was still rising even after 72 h, which is consistent with previous studies [2], suggesting that silane cannot prevent external solutions from intruding when oversaturated. Furthermore, as environmental solutions act as vehicles for aggressive ions, such as chloride ions, from coming into the interior of concrete, low water absorption can also result in a reduction in total chloride ion intrusion and reduce freeze–thaw damage induced by chloride [36,37], as will demonstrated in Section 3.3.

### 3.3. Chloride Ion Penetration Resistance

**Figure 7:**
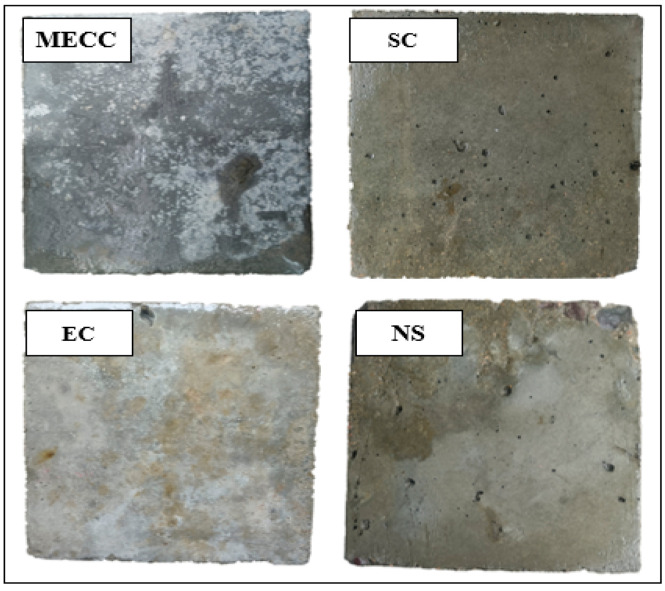
Macro-morphology characteristics of the specimens when exposed to corrosive ions.

In this section, we prove that coating can enhance the chloride ion erosion resistance of concrete by means of fully submerged visual observation and the RCPT test. Figure 7 shows the surface morphology of the four groups of specimens after 120 d of erosion in 10.0 wt% NaCl solution. Visible cracks appeared on the surface of the NS specimen, with visible spalling and aggregate exposure at the corners. No visible erosion can be observed on the surface of any of the coating specimens except for some slight slagging at the corners, which was mostly caused by the molding and repeated handling of the specimens. Spalling of the concrete surface and the resultant production of a large number of cracks will lead to an increase in the intrusion of environmental liquids and corrosive ions, adding to the internal damage of the concrete structure under freezing and thawing [38]. Hence, from an engineering point of view, the EC and MECC specimens are closer to ideal in terms of surface morphology.

Table 5 gives the RCPT results of the four groups of concrete specimens. The average electric flux of the NS specimens was 2128, rated as “medium”. The average electric fluxes of the coating groups were much lower, being 283 (EC), 367 (SC), and 157 (MEEC), which were 13.33%, 17.28%, and 7.39% of that of the NS group. Their ratings were all “very low”. The concrete permeability increased by two grades, suggesting that the three types of coating worked effectively to enhance the chloride ion erosion resistance of concrete. Furthermore, the RCPT results are in good agreement with the water absorption results. The transmission channels inside the MECC specimens were more effectively blocked. The SC group performed the worst among the three coating groups. When oversaturated, SC coating did not effectively prevent external water intrusion [2,3].

### 3.4. Porosity and Pore Size Distribution

**Figure 8:**
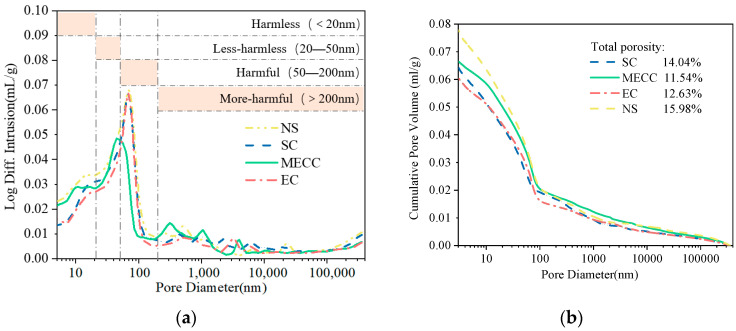
Pore structure of the specimens after coating treatment: (**a**) Log differential intrusion; (**b**) Cumulative intrusion.

The pore structure distribution in the surface layer of the concrete specimens was tested by the MIP test. After the freeze–thaw test, the specimens were taken out and cut to the desired size (10 mm×10 mm × 10 mm). Figure 8 compares the pore structure of the specimens. The total porosities of the NS, SC, EC, and MECC specimens were 15.98%, 14.04%, 12.63%, and 11.54%, respectively. The lower overall porosity of the coated specimens compared with the NS specimens was attributed to the physical barrier provided by the three protectants. However, in terms of pore type, MECC improved the pore structure of cement concrete more remarkably. Concrete pores are categorized into harmless, low-harm, harmful, and high-harm pores. The pore sizes include <20 nm, 20–50 nm, 50–200 nm, and >200 nm [39], as shown in Table 6. Two positive changes can be detected in the internal pore distribution of the MECC specimens. Firstly, the number of >50 nm harmful pores and the proportion of harmful pores reduced. Compared with the NS, EC, and SC specimens, the number of harmful pores (>50 nm) in the MECC specimens was 31.69%, 34.47%, and 48.02% smaller. Secondly, the proportion of 20–50 nm low-harm pores was larger. Compared with the NS, EC, and SC specimens, the number of <50 nm low-harm pores in the MECC specimens was 29.25%, 35.37%, and 28.60% larger, respectively. This indicates that under the same freeze–thaw conditions, MECC greatly prevented the deterioration of the concrete microstructure, reduced total porosity level, increased the number of harmless and low-harm pores that benefit frost resistance, and relieved the damage to the surface structure [38,39]. If additional frost resistance experiments were performed, it is expected that MECC specimens would demonstrate a longer freeze–thaw life than the other groups. For the EC and SC specimens, aside from a smaller proportion of high-harm pores (>200 nm), the pore distribution was similar to that of the NS specimens, aligning with the results described in Section 3.1. After 200 freeze–thaw cycles, the physical barrier in the SC and EC specimens was nearly consumed and their microstructure deteriorated, leaving unevenly distributed pores and cracks on the surface. For the MECC group, as the internal structure was denser, under the same freezing and thawing conditions, the physical protection did not disappear completely. Hence in terms of surface morphology, only part of the MECC specimens was locally damaged by erosion and the other areas remained intact. However, further studies are required to confirm this possibility.

**Figure 9:**
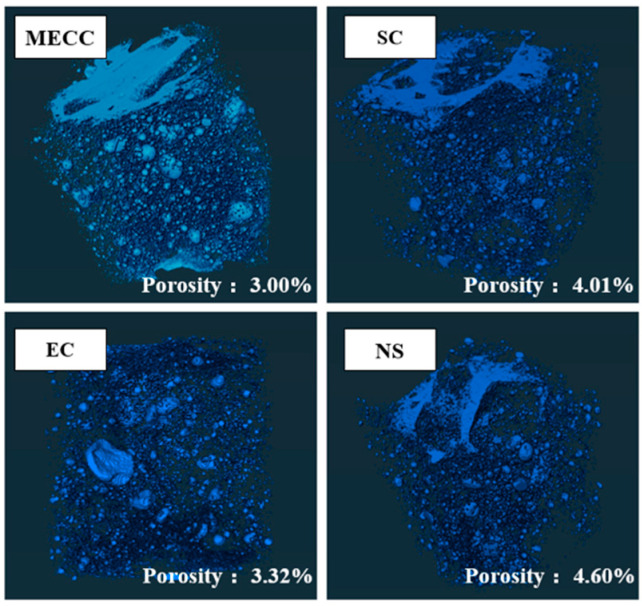
Reconstruction of the CT-scanned inner structure of specimens.

The internal damage of the coated specimens and NS specimens was further determined by CT scanning. These specimens were taken from the concrete’s inner area, about 2–6 cm from the surface. Figure 9 compares the three-dimensional reconstruction images of the internal structure of the specimens. Table 7 provides the pore structure characteristics. After 120 d of salt erosion and 200 freeze–thaw cycles, the total porosities of the internal structures of the NS, SC, EC, and MECC specimens were 4.60%, 4.01%, 3.32%, and 3.00%, respectively. Compared with the result of the MIP test in Figure 8, it can be seen that the freeze–thaw damage to the concrete pore structure mainly occurs at the surface. The porosity of the internal structure can be directly observed in Figure 9. Consistent with the MIP test results, the coating groups had less internal freeze–thaw damage, and MECC provided beneficial protection to the internal structure of concrete.

The CT test data was further divided and grouped into large (≥1.0 mm^3^), medium (0.1–1.0 mm^3^), and small (<0.1 mm^3^) pores. The proportion of small pores was similar among all four groups, measuring 95.05%, 95.48%, 95.95%, and 95.5%. As shown in Table 7, medium pores were low in proportion and small in number. Based on the simple calculation of pore volume = pore number × average volume [40], the four groups did not differ significantly in total number of medium pores, being 644.88 mm^3^, 492.564 mm^3^, 512.63 mm^3^, and 604.31 mm^3^, respectively. Hence, medium and small pores should not be responsible for the density and porosity differences among the specimens. Instead, the greater the freeze–thaw damage inside the structure, the larger the number and proportion of large pores. The proportions of large pores inside the four groups of specimens were 16.55%, 16.8%, 12.14%, and 10.05%, and it is the only group that can be numerically consistent with the total porosity order, confirming that large pores are the main contributor to the difference in average pore volume. In the MECC specimens, the proportion of large pores was 39.27%, 40.17%, and 17.21% smaller than in the other three groups. The total pore volume was approximately half of that in the NS and SC groups and 6.56% smaller than that in the EC group. The MIP and CT results confirm the degree of damage inside the four groups of samples. In light of the results of the analysis in Section 3.1, it is clear that the MECC group had the least internal damage, suggesting that MECC enhances the freeze–thaw durability of concrete.

### 3.5. Mechanism Analysis

The surface hydrophobicity, permeability, and salt erosion resistance of the concrete treated by MECC can be greatly enhanced, effectively reducing the total intrusion of environmental solutions and salts. Due to the physical barrier on the surface of the concrete, when the MECC-treated concrete is water-saturated, its water absorption is reduced when exposed to saturated liquid. According to the theory of porous media mechanics, pore pressure and deformation primarily derive from the hydrostatic pressure induced by volume change during phase transition [35,36]. Hence the degree of damage accumulation is also weakened after repeated freeze–thaw cycles, which can be proved by the MIP test and CT scanning. The MIP results show that MECC effectively increases the density of concrete, which enables the samples to maintain a pore diameter distribution beneficial to frost resistance after freeze–thaw cycles. The pore structure characteristic parameters of the four groups of specimens were tested. The concrete specimens in the MECC group showed the same degree of freeze–thaw damage as we previously expected. They had the best pore distribution on the surface and inside, with the largest proportion of <50 nm harmless pores on the surface and an internal porosity of 56.18% of that of the NS specimens. In addition, due to the surface physical coating, the porosity and average pore diameter of the internal concrete structure were significantly reduced. These findings indicate that specimens treated by MECC can withstand more freeze–thaw cycles and can perform less mass loss and RDEM loss inside before reaching the same critical limit.

## 4. Conclusions

(1) The modified epoxy composite coating (MECC) proposed in this study can significantly enhance the salt frost resistance and the chloride ion erosion resistance of concrete, outperforming ordinary epoxy resin coating (EC) and pure silicate coating (SC). After 150 d of immersion and 200 rapid freeze–thaw cycles, the mass loss of the specimens was only 19.14% of that of the control group, and the RDEM was 31.84% higher compared with the uncoating specimens, which shows good application potential.
(2) The mechanism of the improvement of freeze–thaw resistance was analyzed from the perspective of micropore structure. Under the protection of MECC, the surface hydrophobicity, permeability, and salt erosion resistance of the concrete material are greatly enhanced, and the total intrusion of environmental liquids and salts in the concrete is effectively reduced. This reduces the mass loss and RDEM loss of concrete samples, and the distribution of pore structure is in a state beneficial to the freeze–thaw resistance, leading to an extended freeze–thaw life of concrete.
(3) MECC is an epoxy resin-based surface modifier for cement concrete. This new treatment can help to elongate the maintenance cycle, bring down the maintenance cost, and greatly improve the durability of concrete structures. It has an immersed potential for enhancing the frost and corrosion resistance of concrete pavements. However, there are still some shortcomings in this study. We intend to further investigate the sulfate resistance of MECC-coated concrete and its frost resistance and micro mechanism when exposed to deicing salts, and conduct a scientific evaluation of its environmental friendliness and cost.

## Figures and Tables

**Table 1:** Chemical composition of Portland cement (%).

SiO_2_	Al_2_O_3_	Fe_2_O_3_	CaO	MgO	SO_3_	Na_2_O	f-CaO	C_3_S	C_2_S	C_3_A (C_4_A_3_)	C_4_AF
22.71	4.57	2.85	66.10	1.90	1.37	0.50	0.92	58.06	18.71	5.94	10.82

**Table 2:** Mixing ratio of cement concrete specimens (kg/m^3^).

Cement	Water	Sand	Gravel	Water Reducer (%)
320	129	620	1242	0.8

**Table 3:** Methods of using specimens with different coatings.

Group	Coating Type	Treatment Procedure
NS	No coating	-
EC	Epoxy coating	Apply the first treatment after cleaning the concrete surface, and apply the second after an interval of 5 min; material dosage 0.40 L·m^−2^
SC	Silane coating	Clean the concrete surface and spray three times– in succession; material dosage 0.40 L·m^−2^
MECC	Modified Epoxy Composite coating	Apply three times treatment with a brush after surface cleaning; material dosage 0.35 L·m^−2^

**Table 4:** RCPT evaluation criteria for chloride ion permeability [33].

Electric Flux/C	>4000	2000~4000	1000~2000	100~1000	<100
Chloride ion permeability	High	Medium	Low	Very low	Neglected

**Table 5:** Variations in 6 h coulomb electric flux of specimens.

	NS	EC	SC	MEEC
Electric flux/C	2128	283	367	157
Chloride ion permeability	Medium	Very low	Very low	Very low

**Table 6:** Pore structure results by MIP test.

Group	Proportion of Different Diameter Pores (%)			
	<20 nm	20–50 nm	50–200 nm	>200 nm
NS	28.32%	23.10%	20.81%	27.74%
SC	25.76%	23.60%	24.58%	26.03%
EC	28.76%	23.20%	25.05%	22.97%
MECC	35.96%	30.86%	13.48%	19.68%

**Table 7:** Pore structure test results by CT scanning.

Group	NS	SC	EC	MECC
Porosity (%)	4.60	4.01	3.32	3.00
Average pore diameter (mm)	0.1287	0.1268	0.124	0.1262
Average pore surface area (mm^2^)	0.0222	0.0201	0.0132	0.0137
Average pore volume (mm^3^)	0.1734	0.167	0.1244	0.1273
Pore proportion (<0.1 mm^3^, %)	95.05	95.48	95.95	95.5
Number of pores (0.1–1.0 mm^3^)	2344	1833	1929	2181
Pore proportion (0.1–1.0 mm^3^, %)	0.275	0.268	0.268	0.277
Pore volume (0.1–1.0 mm^3^, mm^3^)	644.88	492.564	512.63	604.31
Number of pores (≥1.0 mm^3^)	361	301	305	334
Pore proportion (≥1.0 mm^3^, %)	16.55	16.8	12.14	10.05
Pore volume (≥1.0 mm^3^, mm^3^)	5974.55	5056.8	3702.7	3356.7

## Data Availability

The original contributions presented in the study are included in the article, further inquiries can be directed to the corresponding author.
